# Editorial: Quantum dots for biological applications, volume II

**DOI:** 10.3389/fbioe.2024.1389974

**Published:** 2024-03-13

**Authors:** Sameer Hussain, Gopinath Packirisamy, Krishna Misra, Mohammad Tariq, Md Palashuddin Sk

**Affiliations:** ^1^ School of Chemistry, Xi’an Jiaotong University, Xi’an, China; ^2^ Nanobiotechnology Laboratory, Department of Biosciences and Bioengineering, Indian Institute of Technology Roorkee, Roorkee, India; ^3^ Department of Applied Science, Indian Institute of Information Technology, Allahabad, India; ^4^ LAQV, REQUIMTE, Departamento de Química, Faculdade de Ciências e Tecnologia, Universidade NOVA de Lisboa, Caparica, Portugal; ^5^ Department of Chemistry, Aligarh Muslim University, Aligarh, India

**Keywords:** quantum dots (QDs), carbon dots (Cdots), Se QDs, biosensing, bioimaging, healthcare, therapy

The use of quantum dots (QDs) which are nanomaterials typically measuring just a few nanometers in size, has revolutionized various research fields over the past decade including bioimaging and therapy. Fascinatingly, the Nobel Prize in Chemistry (2023) is also awarded for the discovery and development of QDs. QDs possess exceptional properties such as remarkable photostability, sensible fluorescence quantum yield (Φ) and a narrow emission band. As a result, they have become preferred over traditional organic fluorophores for a multitude of biological and biomedical applications. Two conventional techniques employed for the preparation of QDs are the bottom-up method and the top-down approach. Furthermore, QDs can be modified with specific ligands known as receptors for targeted applications. In recent years, QDs have showcased immense potential in sensing, imaging, drug delivery, tracking, and phototherapy. Despite various features, the toxicity of most QDs remains a significant challenge for researchers in this field. Therefore, the quest for biocompatible, less-toxic, and brightly luminescent QDs for use in biological systems is a captivating area of research.

This Research Topic aims to highlight and gather the state-of-the art advancements in the synthesis and biomedical applications of QDs. Consequently, researchers were invited to contribute studies focusing on new synthetic procedures, particularly sustainable and greener techniques, and explore their applications in biomedicine. Our Research Topic includes six original research articles, two full review papers, and one minireview, which delve into the synthesis of QDs for various biological applications, and provide insights into emerging trends. Subsequent section provideds a brief overview of the Articles and Review papers covered in the current Research Topic.

Alcoholic gastric ulcer (AGU) is a prevalent and severe gastrointestinal condition. The investigation into the anti-gastric ulcer properties of nanoparticles within the realm of nanomaterials has garnered significant attention from researchers. Kong et al. recently isolated carbon dots (Cdots) designated as FP-CDs, from aqueous extracts of *Fuligo Plantae* and evaluated their therapeutic potential against alcohol-induced gastric ulcer. This was achieved by examining macroscopic images and pathological alterations in gastric tissue, as well as measuring levels of inflammatory and oxidative stress markers. The study not only presents a new approach for exploring the active material component of *Fuligo Plantae*, but also establishes an initial groundwork for the potential clinical use of FP-CDs in treating alcoholic gastric ulcer. In their exciting research, Zhao et al. and co-workers successfully extracted a new type of fluorescent Cdots knows as CMRC-CDs, from *Chrysanthemum morifolium Ramat Carbonisata* using an environmentally friendly method. In murine models, CMRC-CDs were found to alleviate anxiety-like behavior induced by *m*-chlorophenylpiperazine (mCPP) in a dose-dependent manner and demonstrated promising anxiolytic properties. The findings of this study provide valuable insights into how Cdots obtained from traditional herbs could potentially be used to develop innovative, more effective, and safer therapies for anxiety disorders.

Ulcerative colitis (UC) is a bowel disease marked by persistent and progressive inflammation of the intestines. Zhao et al. successfully obtained Cdots, termed as SCR-CDs, from scrambled *Coptidis Rhizoma* using an innovative method. To assess the protective effects against UC, SCR-CDs were tested in a widely accepted mouse model induced by dextran sulfate sodium. The study demonstrated that SCR-CDs, with their diverse surface functional groups, exhibited hygroscopic capacity and hemostatic bioactivity, effectively alleviating key symptoms of UC, particularly bloody diarrhea. This study provides innovative perspectives on how pure plants can be carbonized to prepare Cdots, offering new insights. Furthermore, it holds significant potential in enhancing therapeutic strategies for ulcerative colitis.

Many natural compounds, including naringin, have poor bioavailability due to their low water solubility. Recently, Qu et al. and colleagues acquired Cdots (AFI-CDs) derived from *Aurantia Fructus Immatures*, which demonstrated an impressive solubilization effect, increasing the solubility of naringin by a factor of 216.72. This study provides fundamental insights into the solubilization mechanism of naringin using AFI-CDs, offering a new approach to address the difficulties linked with the low water solubility of drugs that are insoluble in water in the field of modern pharmaceutical science.

Liver fibrosis is a transitional phase in the advancement of liver disease, and currently, there is no recognized clinical therapy available for effective treatment of fibrosis. In their subsequent research, Qu et al. and colleagues acquired biocompatible Cdots (VSC-CDs) from *Vaccaria Semen Carbonisatum* using a one-step pyrolysis technique. Spherical shaped and negatively charged VSC-CDs exhibit a quantum yield of ∼2.08%, with diameters in the range 1–5.5 nm. VSC-CDs, possessing ample chemical functional groups and excellent water dispersibility, not only improved liver function and mitigated liver damage in pathomorphology but also reduced the severity of liver fibrosis. The results of this study offer potential for the advancement of a new, eco-friendly, and efficient nanomedicine, providing a promising therapeutic approach for attenuating liver fibrosis in clinical settings.

The lack of vitamin D is linked to a range of conditions including osteoporosis, obesity, depression, digestive Research Topic, and infections. Consequently, it has become a subject of sinificant interest in the field of healthcare. Yue et al. developed a fluorescence immunochromatographic assay based on ZnCdSe/ZnS QDs for the prompt, visual and quantitative detection of 25⁃hydroxyvitamins D (25-OF VD) in human serum. The assay demonstrated good selectivity and a linear detection range of 5–100 ng/mL for 25-OH VD in phosphate buffer saline. This study represents a pioneering application of QDs for fast, visual and quantitative identification of 25-OH VD, holding significant promise for the clinical diagnosis of diseases linked to vitamin D.

Despite substantial advancements in research, cancer continues to be a highly fatal disease on a globe scale. The Review paper published by Omer et al. in this Research Topic magnificently summarizes the recent advancements in the usage of group-11 (Cu, Ag and Au) Cdots regarding their potential for early cancer diagnosis and therapy, which includes their nanohybrids, nanocomposites, heterostructures, and more. It specicially emphasizes the role of Cu-, Ag-, and Au-doped Cdots as nanotheranostic agents for cancer treatment in terms of imaging applications (e.g., fluoresecnce, photoacoustic, magnetic resonance imaging) and therapeutic applications (e.g., photodynamic, photothermal, multimodal). Based on extensive research, it is believed that Ag-, Cu- and Au-doped-Cdots are new emerging class of C-based fluorescent nanomaterials for cancer theranostics.

Mondal et al. highlighted the wide-ranging applications of Cdots in their Review paper, including bioimaging, gene therapy, phototherapy, medication delivery, and more. The initial section of the paper discussed various synthetic techniques, as well as the structure and properties of Cdots. The subsequent sections focused on recent advancements, future prospects, and multiple applications of Cdots and their composites, as well as hetero-atom-doped modifications, specifically in the field of bioimaging and therapy.

Selenium quantum dots (Se QDs) is a new type of fluorescent nanomaterial that provide various advantages including ultra-small particle size, distinct optical/surface properties, and low-toxicity. Consequently, Se QDs have become a promising player in the realm of non-metallic luminescent QDs, making them highly suitable for a wide range of biological applications. The sole Minireview published by Li Zhou et al. on this Research Topic elegantly summarizes preparation of various fluorescent Se QDs for biomedical applications such as detection of biomolecules, cell/tissue imaging and therapy. The review also discusses the existing challenges and future prospects of Se QDs in the domain of biosensing and bioimaging.

In summary, this Research Topic offers recent updates on biocompatible QDs derived from various plant sources using greener synthetic methods, highlighting their potential for diverse biomedical applications. These applications encompass the treatment of alcohol-induced gastric ulcers, anxiety disorders, ulcerative colitis, liver fibrosis, and diseases associated with vitamin D. The Review papers collected on this Research Topic also revealed that QDs represents a promising class of nanomaterials in the field of healthcare. Indeed, Se QDs has garnered substantial attention as an emerging type of fluorescent QDs in recent years. However, their low photostability when continuously exposed to excitation light poses a significant challenge and can greatly impact their fluorescence properties. Addressing this Research Topic remains a crucial and ongoing task in the field. Additionally, the challenges of cost-effective synthesis and large-scale industrial production of QDs are also significant hurdles that need to be addressed in the near future. Besides, the primary challenge for biomedical scientists persists in the form of the low toxicity and inadequate biocompatibility of most QDs. These limitations curtail the potential applications of QDs in biomedicine. Furthermore, the limited capability of numerous QDs to penetrate the blood-brain barrier poses a significant obstacle to their use in targeting brain tumors. Therefore, further research is warranted on the preparation of QDs with reasonable biocompatibility, good photostability, easy synthetic methods, and the ability to penetrate cells.

